# Editorial: Branching and Rooting Out with a CT Scanner: The Why, the How, and the Outcomes, Present and Possibly Future

**DOI:** 10.3389/fpls.2016.00041

**Published:** 2016-02-02

**Authors:** Pierre Dutilleul, Jonathan A. Lafond

**Affiliations:** ^1^Department of Plant Science, McGill UniversityMontréal, QC, Canada; ^2^Département des sols et de Génie Agroalimentaire, Université LavalQuébec, QC, Canada

**Keywords:** X-ray computed tomography (CT) scanning, leaf canopies and branching patterns, root systems and other below-ground plant organs, graphical vs. quantitative and statistical analyses, summary and perspectives on plant CT scanning

At the time this editorial was written, an extremely interesting loop was closing! Indeed, this text was written last after the invitation received in October 2012 and the proposal submitted in August 2013 for a *Frontiers in Plant Science* research topic (specialty: Plant Biophysics and Modeling) on “plant CT scanning,” the preliminary submission of abstracts in December 2013 and the seven peer-reviewed contributions endorsed for publication and published in March-December 2015. A rapid “guided tour” of the Research Topic, finally titled “Branching and Rooting Out with a CT Scanner” (sub-title: “The Why, the How, and the Outcomes, Present and Possibly Future”), is proposed here. The field of research (excluding synchrotron-based X-ray microtomography of plant tissues) is introduced first, followed by the aims and scope of the Research Topic and a brief description of the major achievements made in each of the articles (2 Methods, 4 Original Research, 1 Perspective). Acknowledgements are made separately.

The use of X-ray computed tomography (CT) scanning with plants is part of the incorporation of new technologies into the scientific research work. Since CT scanners were originally designed for medical applications and later for industrial applications, some adjustment to the reality of the plant world has been necessary, in the air or in the soil or water medium depending on the plant structure or material of interest: the branching pattern in a leaf canopy and the leaves themselves, or a root system. To the best of our knowledge, the first published CT scanning application in which plants were involved is the study of Tollner et al. (1987). The plant part of it consisted in visualizing portions of a root system in the surrounding soil biota, through a few 2-D cross-sectional CT images with no attempt to isolate the entire root system from the soil medium and produce a 3-D image. Plant CT scanning applications to study leaf canopies and relate the complexity of branching patterns to light interception in particular came only in the early 2000s (Dutilleul, 2003), but immediately with 3-D image construction because a canopy is surrounded with air, the challenge coming from the separation of leaves from branches.

Until recently, a majority of the applications of X-ray CT scanning in plant sciences remained descriptive; some included a quantification of plant materials when the root-soil isolation or branch-leaf separation was satisfactory; and a few involved the modeling of plant biology processes or the assessment of treatment or disease effects on plant biomass and structures during growth. In the last decade, repeated CT scanning of the same plants was reported in an increasing number of studies in which moderate X-ray doses had been used. Besides the general objectives of *Frontiers in Plant Science* research topics, “Branching and Rooting Out with a CT Scanner” was proposed to meet specific objectives: (i) providing a non-technical update on knowledge about the application of CT scanning technology to plants; (ii) drawing the limits of the CT scanning approach, which is based on material density; (iii) explaining with a sufficient level of detail the main procedures used for graphical, quantitative, and statistical analyses of plant CT scanning data, including fractals and statistics appropriate for repeated plant CT scanning, in experiments where the research hypotheses involve key biological processes; (iv) comparing plant CT scanning with an alternative technology targeting leaf canopies; and (v) providing current and potential users of plant CT scanning with up-to-date information and exhaustive documentation, including clear perspectives and well-defined goals for future projects.

Starting with the two Methods articles, Dutilleul et al. and Vello et al. can be used to briefly discuss objectives (ii)–(iv). On the plant CT scanning side (Dutilleul et al.), the crowns of various miniature conifers with contrasting leaf types (needlelike vs. scalelike) were reconstructed in 3D from the CT numbers collected at high resolution, and the crown traits measured from 3-D images (leaf areas and volumes, fractal dimensions of branching patterns) were correlated with a shade tolerance index, the conclusion being that mean values and correlations can differ with leaf type. Regarding the use of phenomics platforms with plants (Vello et al.), alternative non-invasive technologies, exploiting sensors for visible, fluorescent and near-infrared lights, are described to accurately screen survival phenotypes in *Arabidopsis thaliana* exposed to water-limited conditions. Following two drought protocols and a robust analysis methodology, the authors clearly assessed the plant wilting or dryness status at different time points. In the Original Research articles by Subramanian et al. and Sturrock et al., root system architectures of crop seedlings were investigated, respectively, under salt stress over weeks following seed planting (crop: corn) at high resolution and over days after inoculation of a plant pathogenic fungus (crops: wheat, oil seed rape) at ultra-high resolution, both using repeated-measures ANOVA for statistical analysis because of the repeated plant CT scanning. While Subramanian et al. included fractal dimensions of upper- and lower-root systems of corn seedlings among the measured traits, Sturrock et al. assessed disease severity in relation to pathogen DNA quantification in soil using real-time PCR. Very interestingly, Paya et al. used X-ray CT scanning to uncover root–root interactions and quantify spatial relationships between interacting root systems in 3D. More specifically, they assessed the effects of inter- vs. intra-specific interactions of very young tree seedlings (quaking aspen, black spruce) on their development and utilization of 3-D space by roots, with adjustment of the statistical analysis to the unbalanced design. Very originally, Koebernick et al. combined soil and root water flows by simulations depending on the parameterization and description of the root system (crop: faba bean), and showed how developing root architectures derived from temporally repeated X-ray CT scanning can be implemented in numerical soil-plant models. With their Perspective article. Lafond et al. provide a timely summary of the situation in the X-ray CT scanning of root systems and leaf canopies, data collection and analyses, thus fulfilling objectives (i) and (v) of the Research Topic and helping readers to be efficient in their future work.

## Author contributions

For this Editorial, PD prepared a first draft, which was read and edited by JL before and after a meeting in Montréal, Canada on Wednesday, December 16, 2015. The submitted version is the result of the final editing by PD following JL's comments. Including the Editorial, PD signs/co-signs a total of four contributions, while JL signs/co-signs two. As Topic Editors, PD handled and coordinated the review of three of the other submissions that were endorsed for publication, and JL, one.

### Conflict of interest statement

The authors declare that the research was conducted in the absence of any commercial or financial relationships that could be construed as a potential conflict of interest.
